# Co-designing implementation strategies for social prescribing in Lancashire and South Cumbria: a qualitative study with a participatory approach

**DOI:** 10.1136/bmjopen-2024-094522

**Published:** 2025-04-02

**Authors:** Sima Rafiei, Mahsa Honary, Barbara Mezes, Susan Flowers

**Affiliations:** 1 Department of Management Science, Lancaster University Management School, Lancaster, UK; 2 Department of Primary Care & Mental Health, Institute of Population Health, University of Liverpool, Liverpool, UK; 3 Rural & Social Development Through the Arts, Green Close Ltd, Lancaster, Lancashire, UK

**Keywords:** QUALITATIVE RESEARCH, Community-Based Participatory Research, Psychosocial Intervention, Health policy

## Abstract

**Abstract:**

**Objectives:**

Social Prescribing (SP) programmes hold significant promise, yet there is a critical need to identify the underlying causes of their challenges and develop evidence-based, co-designed solution ideas through a collaborative approach.

**Design:**

This study applied a multimethod participatory design using co-design workshops to generate solutions to root problems and a 2-day Citizen Jury (CJ) event to validate these solutions from different perspectives.

**Participants:**

Four co-design workshops were conducted with different stakeholders, including experts-by-experience, community providers, SP link workers and other health and social care professionals who were responsible in SP coordination and leadership.

**Data analysis:**

Data were analysed using thematic analysis, identifying the root causes across several domains: human resources issues, social determinants of health), intrasectoral and intersectoral collaboration in health planning and service delivery, knowledge and awareness, financing, assessment systems for evaluating SP programme effectiveness, information systems and supportive policies/mechanisms.

**Results:**

11 solutions were proposed, including prioritising a human-centric approach, establishing sustainable funding mechanisms, providing sufficient training and knowledge for staff, fostering co-production and shared vision across sectors, adopting a preventive approach to healthcare, enhancing information system support and encouraging self-referrals. These strategies were proposed and refined during the 2-day CJ event.

**Conclusions:**

Using a participatory method enabled a comprehensive understanding of different stakeholders’ perspectives and facilitated the development of co-produced solutions based on the identified challenges. This approach has the potential to assist policymakers in developing realistic policies to enhance social care within integrated care systems.

**STRENGTHS AND LIMITATIONS OF THIS STUDY**

- This study employed a participatory co-design approach, ensuring that the solutions proposed were informed by the experiences and perspectives of diverse stakeholders, including experts-by-experience, social prescribing link workers and healthcare professionals.
- The use of a multimethod approach, including co-design workshops and a Citizens’ Jury event, allowed for both the generation and validation of solutions, enhancing the credibility and relevance of the findings.
- The incorporation of both qualitative and consensus-based methods strengthened the depth and reliability of the data collected.
- The study was conducted within Lancashire and South Cumbria, which may limit the generalisability of the findings to other regions with different social prescribing structures and healthcare systems.
- While the study identified implementation challenges and potential solutions, it did not evaluate the practical application or long-term effectiveness of these strategies in real-world settings.

## Introduction

 Despite growing recognition of social prescribing (SP) as a strategy for improving health outcomes, its implementation remains complex and challenging. SP aims to connect individuals with non-medical, community-based services to address social determinants of health (SDH), yet significant structural and operational barriers hinder its widespread success.^1^ These challenges include inconsistent funding, workforce shortages, ineffective cross-sector collaboration and fragmented referral systems.^2^ While previous research has documented these obstacles, few studies have investigated the root causes underlying them, leaving gaps in understanding how to create sustainable solutions.^3 4^ Without addressing these deeper, systemic factors, interventions remain reactive and may fail to generate long-term improvements.^5^

To bridge this gap, this study adopts a root cause analysis (RCA) approach, combined with a co-design methodology, to identify and address fundamental implementation barriers in SP programmes. RCA has been widely applied in healthcare implementation science to move beyond surface-level challenges and pinpoint structural, operational and systemic issues affecting service delivery.^6^ Alongside this, co-production—a participatory process involving stakeholders such as experts-by-experience (EbEs), service providers and policymakers—is used to develop evidence-based, contextually relevant solutions.^7^ Co-production facilitates stakeholder engagement, increases intervention acceptability and enhances real-world feasibility. Prior studies indicate that co-production enhances the acceptability and effectiveness of interventions by prioritising stakeholder-driven solutions.^8^

Furthermore, this study is framed within the broader context of knowledge translation (KT), which emphasises the process of transforming research findings into actionable strategies for implementation. Despite growing evidence on SP, a critical gap remains in translating these insights into sustainable, stakeholder-driven interventions. Co-production serves as an effective KT approach by fostering active collaboration between researchers, service users and practitioners, thereby ensuring that solutions are relevant, feasible and implementable.^7^ We applied knowledge transfer through adhering to the following principles:

- **Engagement and exchange:** stakeholders (eg, policymakers, practitioners, community representatives) actively contributed throughout the research process.
- **Contextual adaptation:** rather than assuming a top-down implementation model, we facilitated ongoing dialogue to ensure that solutions were contextually relevant and feasible.
- **Sustainability considerations:** by fostering stakeholder ownership and capacity-building, we aimed to enhance the long-term viability of the co-designed strategies.

While numerous studies have identified the challenges in implementing SP, these challenges persist despite repeated efforts to mitigate them. Addressing these issues without systematically analysing their root causes risks implementing short-term solutions that may not resolve the underlying structural, systemic and operational barriers.^9^

Thus, a RCA approach is crucial as many implementation failures stem from deep-seated, inter-related factors that go beyond the visible obstacles. The need for RCA is well-documented in complex system interventions.^10^ In healthcare, solutions that are designed without understanding the systemic origins of problems often lead to unintended consequences.^11^

This study argues that merely addressing known SP challenges without understanding their root causes is insufficient. Instead, we propose a structured, participatory approach that engages diverse stakeholders including service providers, link workers (LWs) and EbEs to diagnose underlying factors and design contextually relevant, co-produced solutions that ensure the long-term sustainability of SP initiatives. This root cause-based approach aligns with best practices in implementation science and systems-thinking frameworks, both of which emphasise the need to identify causal relationships and upstream determinants before designing interventions.^12^

This article addresses this gap by applying a co-design approach to generate evidence-based, stakeholder-driven strategies for addressing the implementation challenges of SP programmes. Specifically, this study seeks to answer the following research question: what are the root causes of the key challenges in implementing SP programmes, and how can co-designed strategies be developed to address these challenges effectively?

To structure the co-design process, we reviewed multiple methodological frameworks and selected the Hasso Plattner Institute of Design’s five-step approach (define, ideate, prototype, test and implement) due to its iterative nature, stakeholder-driven focus and alignment with participatory research principles. This framework was preferred over traditional linear models as it allowed for continuous stakeholder input and solution refinement throughout the research process.^13^

## Methods

### Study design

This study applies participatory research methods; however, it is primarily academically driven, with researchers initiating the study design and then incorporating participatory approaches to engage key stakeholders. Specifically, co-design workshops and a Citizens’ Jury event were used to facilitate collaboration between SP LWs, community providers (CPs), EbEs and policymakers. These participatory elements were introduced to ensure that stakeholders played an active role in problem-solving and developing actionable solutions, rather than merely being research subjects. However, the initial conception and methodological framework of the study were developed by the research team.

### Participants

Four co-design workshops were conducted, each involving 6–7 participants rather than the same 24 participants attending every session. The workshops were structured to include a diverse mix of stakeholders, ensuring representation from EbEs, LWs, CPs and healthcare professionals across different sessions. Accordingly, we adopted a purposive sampling strategy by targeting specific stakeholder groups. We deliberately varied the composition of participants across the four workshops, ensuring that each session included a mix of these stakeholder groups. This approach allowed us to incorporate a wide range of perspectives while also accommodating participants’ availability and expertise. This approach enabled a broad range of perspectives to be incorporated into the co-design process while allowing participants to contribute based on their availability and expertise.

The inclusion criteria included (1) a minimum age of 18 years old and (2) having experience with SP provision. Those not actively involved in the SP programme or its coordination as well as individuals who were unable to provide informed consent due to cognitive impairments or language barriers were excluded from the study. Furthermore, participants who could not commit to the full duration of the co-design workshops or the 2-day Citizens’ Jury event were not included in the study.

In our study, displaying posters and flyers in public venues such as general practitioner (GP) surgeries, community centres, libraries and locations where SP activities were offered formed the primary means of recruitment. However, this was supplemented by more active outreach methods to ensure a broader and more engaged participant pool. Specifically, the National Institute for Health and Care Research supported us during the recruitment phase. This collaboration helped increase visibility and reach, facilitating engagement with a wider pool of potential participants.

### Study procedure

Potential participants were encouraged to contact the study team by phone or email. Following informed consent, participants completed a demographic questionnaire and signed up for the co-design workshops. During the workshop, participants were presented with some of the identified challenges of SP.^14^ Subsequently, four sessions of co-design workshops were held at Lancaster University, UK, between September and October 2023.

### Research team

Our research team included experts in public health and policy, social care, design thinking, psychology and qualitative research with extensive experience in participatory research and community engagement, ensuring a structured yet flexible approach to co-design.

### Workshop structure and setting

The workshops were staged at the School of Management Science, Lancaster University, between September and October 2023. Sessions were held in both the morning and afternoon to accommodate diverse participant schedules. Each workshop brought together a multidisciplinary group, including GPs, LWs, representatives from the VCSE (Voluntary, Community, and Social Enterprise) sector, policymakers and individuals with lived experience. To create a collaborative and inclusive environment, we employed structured co-design techniques such as 5 Whys and Crazy 8s.^13 15^ These approaches encouraged deep problem exploration and rapid idea generation, ensuring all participants could contribute meaningfully.

A range of participatory research methodologies were considered to support the co-design process, including the Experience-Based Co-Design framework,^16^ Integrated Knowledge Translation approaches^17^ and Community-Based Participatory Research.^18^ In this study, the Hasso Plattner Institute of Design’s five-step approach was selected due to its problem-solving nature, being in line with the complexity of SP implementation. This approach contains five steps, including ‘empathise, define, ideate, prototype and test’ which allows for continuous refinement of solutions based on stakeholder feedback, ensuring that the proposed strategies were both user-centred and feasible.^13^

Each co-design workshop lasted 2 hours and was structured into five sections. In each of these sections, we progressively developed and implemented the five steps of the HPI (Hasso Plattner Institute) Design Thinking methodology, ensuring that participants actively contributed to each phase.

### How workshops were structured in five distinct sections

Here, we explain the breakdown of how the methodology was applied in each section of the workshops:

- Empathise phase (section 1): In the first section of each workshop, we focused on understanding participants’ experiences with SP. Participants shared their perspectives, challenges and personal experiences, allowing us to gain valuable insights into the current issues surrounding SP. This step was critical in building a shared understanding of the problems participants faced.
- Define phase (section 2): The second section of each workshop focused on synthesising the information gathered during the empathise phase. We facilitated group activities, such as affinity mapping, to help participants organise and identify common themes. Together, participants defined the key problems that would form the focus for the co-design process. This stage helped ensure clarity on the challenges we aimed to address.
- Ideate phase (section 3): In the third section, participants engaged in ideation activities, brainstorming potential causes of the problems defined in the previous phase. Using creative techniques like fishbone diagrams helped identify potential causes contributing to each problem. The facilitator guided participants in applying the 5 Whys method to uncover the root causes for each challenge. Challenges were categorised with the facilitators’ assistance. The ideas were discussed and refined within groups to foster innovative solutions that could be applied in the context of SP.
- Prototype phase (section 4): The fourth section of each workshop was dedicated to prototyping. Here, participants worked in small groups to develop low-fidelity prototypes of their ideas. These prototypes were designed to be simple, quick representations of potential solutions, allowing participants to visualise and communicate their concepts. To do so, the Crazy 8 method was used, and participants were prompted to generate as many new and creative ideas as possible within a structured timeframe. They were given two questions derived from the root causes identified in the previous phase and were encouraged to propose eight solutions to each question in just 8 min. Feedback was gathered from the group to improve the ideas further.
- Test and reflect phase (section 5): In the final section of each workshop, participants discussed how they would work in real-world settings and reflected on their feasibility. Feedback from both group discussions and individual reflections helped refine the prototypes and further solidified the application of the solutions.

Throughout the workshops, we ensured that participants from diverse backgrounds (EbEs, SP LWs, CPs and healthcare professionals) contributed to each stage of the process, ensuring a broad range of perspectives in every phase. By organising each workshop into these five sections, we were able to cover all phases of the co-design process iteratively, ensuring that each step was integrated into every session. This structure provided participants the opportunity to move through each stage of the HPI Design Thinking methodology and contribute meaningfully to the development of solutions for SP.

### Citizen Jury

Finally, to validate the proposed solutions, a 2-day Citizen Jury event was organised. The CJ process was structured over 2 days, with the following key stages:

1. **Preparation andrecruitment**: Citizens were selected through a recruitment process that aimed to ensure a broad range of perspectives, including individuals from varying socio-economic backgrounds, ages and experience with SP. Accordingly, we recruited 32 participants via Eventbrite, including (1) 15 EbEs as jurors; (2) 5 experts, including 2 CPs, 1 EbE, 1 health and social professional and 1 LW; and (3) 12 guests consisting of CPs, LWs, National Health Service (NHS) staff and policymakers, including a director of public health. Delegates received an agenda (see online supplemental file) before the event.
2. **Initial information and framing (day 1)**: The first day involved providing participants with background information on the SP challenges. Experts, including healthcare professionals and community leaders, delivered presentations to inform the group about the current state of SP, the challenges faced and the potential for improvements. Following these presentations, participants were given time to ask questions and engage in group discussions to better understand the issues.
3. **Deliberation anddiscussion (day 1afternoon andday 2morning)**: On day 1 afternoon and day 2 morning, participants engaged in deliberative discussions in smaller groups. Each group was tasked with reflecting on the information provided, identifying key issues and considering potential solutions to the challenges of SP. The group discussions were facilitated to encourage equal participation and ensure diverse perspectives were represented.
4. **Developingrecommendations (day 2afternoon)**: On the final day, the participants collaboratively worked to draft a set of recommendations based on their deliberations. These recommendations focused on actionable improvements to SP processes, policy suggestions and co-designed solutions that could be applied to real-world contexts.
5. **Presentation andconclusion**: At the end of the second day, each group presented their recommendations to the larger Citizen Jury, followed by a discussion to refine and consolidate the final recommendations.

To analyse the findings from the Citizen Juries, we used a combination of qualitative analysis methods:

- **Thematic analysis**: We transcribed the discussions from the Citizen Juries and employed thematic analysis to identify recurring themes, concerns and ideas across the different groups. This involved coding the transcripts and grouping similar points together to develop broader themes that captured the essence of participants’ views on SP.
- **Synthesis with workshop findings**: The findings from the Citizen Juries were then compared and synthesised with the results from the four co-design workshops. The aim was to assess how the perspectives from citizens (Citizen Jury participants) aligned or differed from those shared by stakeholders in the workshops. This comparative analysis allowed us to draw comprehensive conclusions about potential solutions and priorities for improving SP.
- **Recommendations distillation**: The final step of the analysis focused on distilling actionable recommendations from the Citizen Jury discussions. These recommendations were categorised into themes based on their relevance to policy, practice and system-level improvements. This phase of the study allowed for independent critiques and the development of a final consensus of participants.

### Data analysis

Audio data were collected throughout the workshops and the CJ. Data were transcribed and entered into the MAXQDA software V.2020. To enhance the quality and rigour of the thematic analysis reported in the study, a coding framework was developed at the outset to guide the analysis. This framework was informed by the research questions and the key themes emerging from the data. We used both deductive (predefined categories) and inductive (emerging patterns from the data) approaches to coding, ensuring flexibility while maintaining focus on the research objectives. Furthermore, throughout the coding and analysis process, regular discussions were held among the research team to review the emerging themes and ensure a shared understanding of the data. These discussions provided an opportunity for researchers to challenge assumptions, consider alternative interpretations and refine the thematic framework. This process helped enhance the rigour of the analysis and allowed us to address potential biases.

Third, in order to further validate the themes and interpretations, we conducted a process of member checking. Selected participants from the workshops and Citizen Juries were invited to review and provide feedback on the preliminary findings and themes. This ensured that the themes accurately represented their experiences and perspectives, enhancing the credibility of the analysis.

### Patient and public involvement

This study involved active engagement with a diverse range of stakeholders, including EbEs, CPs, LWs and health and social care professionals. The co-design workshops and the Citizens’ Jury event were designed to facilitate collaboration between these groups to ensure that the solutions developed were grounded in the real-world experiences of individuals directly involved in SP. The input from the public and patients was central to the development of the implementation strategies, allowing for a participatory approach that reflected the needs, perspectives and priorities of those who would ultimately be affected by the changes. These stakeholders contributed to identifying root challenges, proposing solutions and refining the strategies through feedback during the study.

## Results

Participants were first asked to revisit the key challenges identified in the first phase of the research, including (1) financial issues and sustainability, (2) human resources challenges, (3) partnership working challenges, (4) inadequate and inconsistent implementation, (5) information system challenges, (6) referral system issues, (7) training and knowledge gaps and (8) accessibility and privacy concerns.

### Identifying the underlying causes of challenges

**Figure 1:**
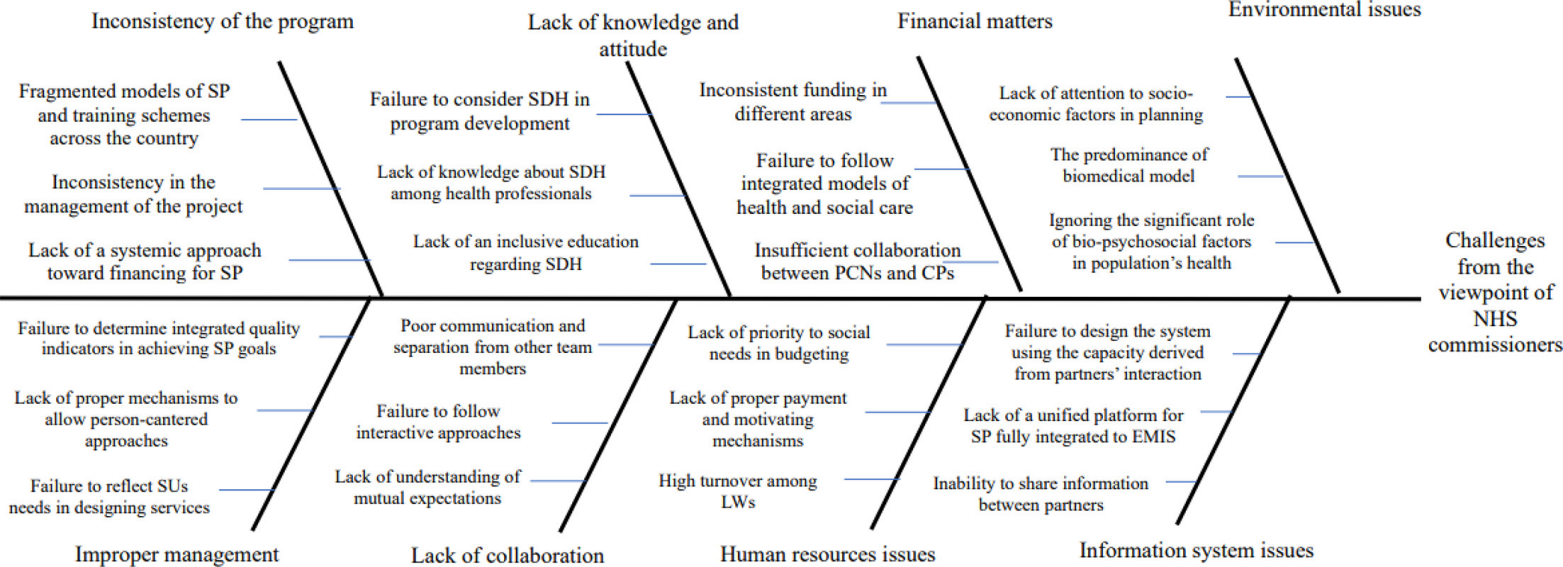
The root causes of challenges from the viewpoint of NHS commissioners. CP, community provider; EMIS, Emergency Medical Services Information System; LW, link worker; NHS, National Health Service; PCN, primary care network; SDH, social determinants of health; SP, social prescribing; SU, Service users.

Figures1^4^ represent the fishbone diagram for the challenges mentioned by different stakeholders of the study. Using the diagram, several causes are contributing to the identified challenges, each with equal importance in the fish bone diagram. According to health and social care professionals, the following root causes contributing to these challenges were ignoring the significant role of bio-psychosocial factors in population’s health policymaking, insufficient collaboration between primary care networks (PCNs) and CPs, lack of academic training regarding SDH, absence of a systemic approach towards financing for SP, failure to design a system that harnesses capacity from partners’ interactions, lack of prioritisation of social needs in budgeting, poor communication and isolation from other team members, and failure to determine integrated quality indicators to achieve SP goals (figure 1).

**Figure 2:**
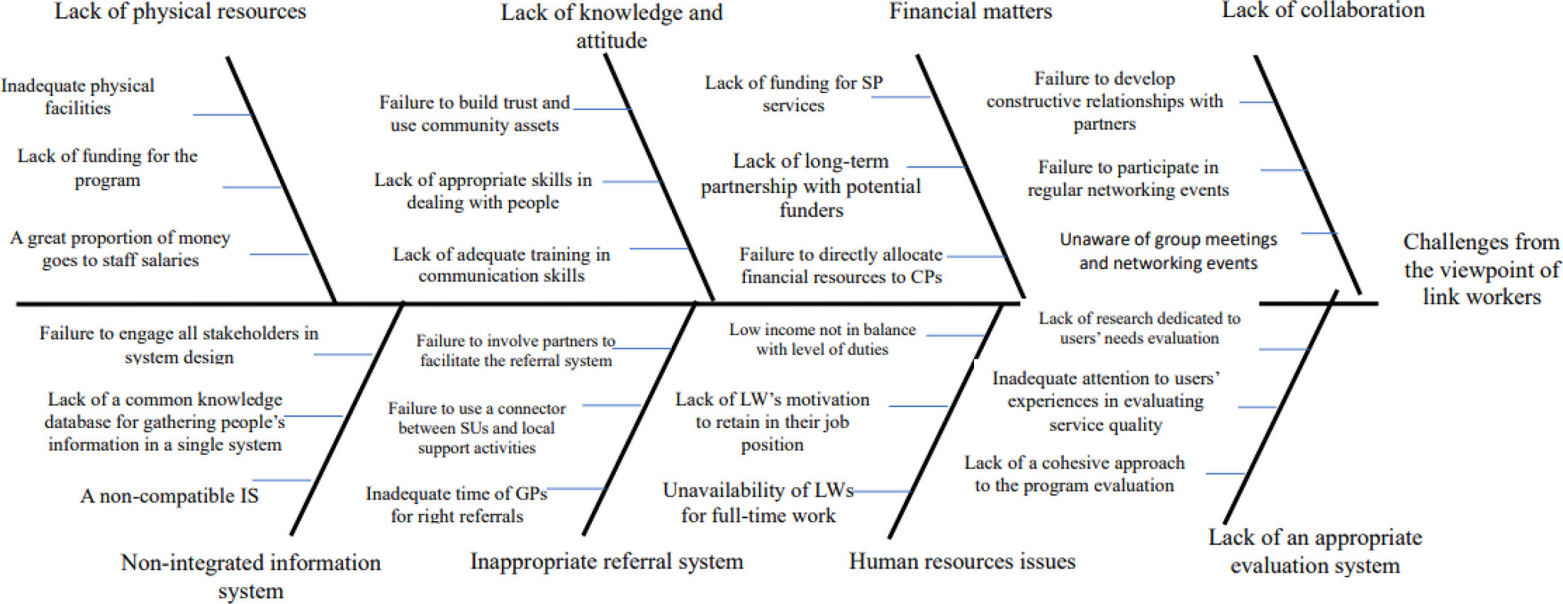
The root causes of challenges from the viewpoint of link workers. CP, community provider; GP, general practitioner; LW, link worker; SP, social prescribing.

**Figure 3:**
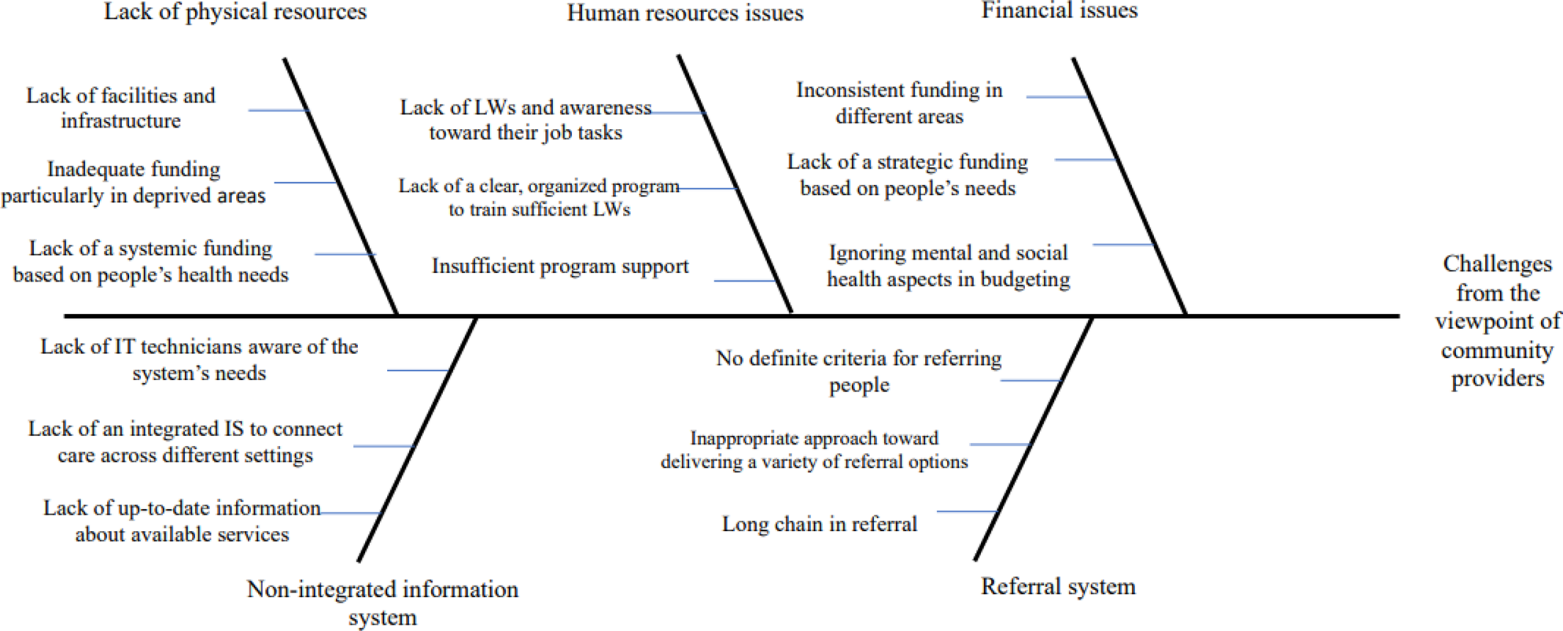
The root causes of challenges from the viewpoint of community providers. IT, information technology; LW, link worker.

**Figure 4:**
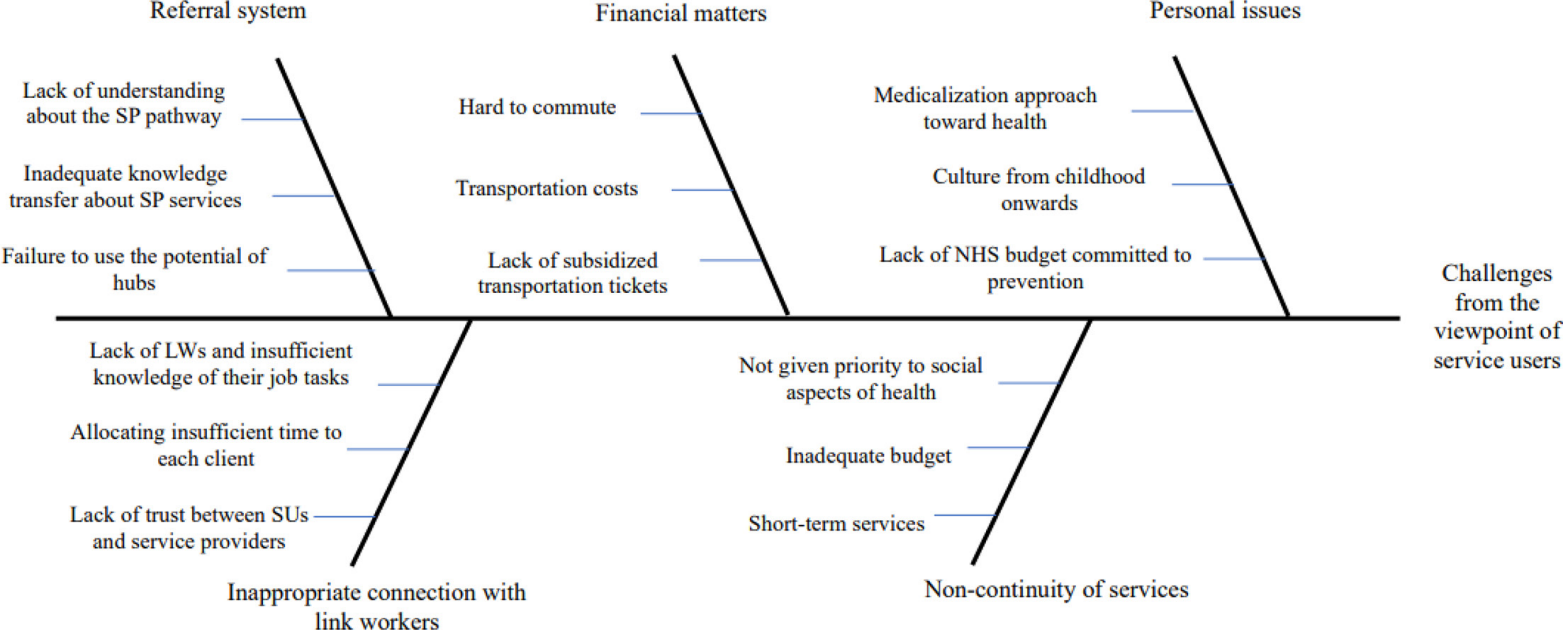
The root causes of challenges from the viewpoint of service users. LW, link worker; NHS, National Health Service; SP, social prescribing; SU, Service users.

According to the LWs’ opinion, the root causes of challenges were as follows: a failure to directly allocate financial resources to CPs, lack of adequate training in communication skills, the directing of a great proportion of money towards staff salaries, being unaware of group meetings and network events, low income not in balance with LW levels of duties, a failure to involve partners in facilitating the referral system, a failure to engage all stakeholders in system design and lack of research projects dedicated to evaluating users’ needs.

CPs mentioned that ignoring mental and social healthcare aspects in budgeting, insufficient support of community well-being activities, lack of a systemic funding, the absence of definite criteria for referring people to SP services and the lack of IT technical support were the root causes of the following problems: resource management, implementation of the referral system and users’ information management.

Finally, EBEs contributed that the lack of NHS budget dedicated to preventive care, the lack of support policies to cover transportation costs, a failure to use the potential of hubs, an inadequate attention to social aspects of health and lack of LWs and insufficient knowledge about their tasks were the most fundamental causes of challenges in the SP system.

### Suggested strategies for improvement

A combination of results obtained from the co-design workshops and the Citizen Jury event led to 11 themes to resolve the challenges.

### Macro-meso-micro framework for thematic analysis

- **Macro level (systemic and policy context):** The macro level focuses on broader, system-wide factors, such as policy, funding and governance, which shape the implementation of SP. Accordingly, the themes are as given below.

#### Theme 1: adequate funding for SP

According to study participants, there should be adequate funding for preventive care to tackle the SDH. They underlined the need for long-term funding mechanisms that promote constructive relationships with EbEs. *“I’m worried about community providers especially those working in small organisations*” (LW3). To resolve the issue, some of the CPs noted that funding resource needs to be directly provided to the third sector. “*So, what I’m looking to do is put money into the third sector to deliver that friendly service, all-inclusive friendly service*” (CP2).

Using the potential of the private sector was another strategy to support financing for SP activities. Stakeholders emphasised that many private sector entities are interested in sustaining partnerships with CPs. They suggested that adopting a competitive strategy could incentivise funders to increase investment in social services and integrated approaches to health and social care services. *“Here, we’ve got the private sector, well, we don’t need to go into them, that’s all your businesses, and that’s your money makers”* (CP4).

Health and social care professionals suggested a range of combined funding approaches with maximum collaboration across sectors and shared responsibilities toward funding for local services. “*Local government, NHS, social care, and private sector should work together and provide all the funding”* (NHS4). Some participants highlighted the significant role of local authorities in providing central grants to support social care services. They explained that a dedicated tax to fund SP services could be an evidence-based funding strategy to cover vulnerable people in receiving basic social services. “*Yes, can we have national funding sectors or something, national funding bodies?”* (CP1). While others disagreed with this proposed mechanism and mentioned it as a barrier for sustainable funding. They also expressed concerns about equity of access to SP services, noting potential disparities in the quality of services provided by different providers. Instead, they emphasised that implementing financing methods that would require CPs to improve waiting times and deliver superior, and sustainable services would be a viable solution. “*Improvement to the productivity of service delivery is a good criterion for budgeting”* (NHS2).

Some CPs acknowledged that they need clearly defined strategies from funders. *“Whatever the funding is; usually, you would have the strategy before you apply for the funding”* (CP2).

Service users believed that funding for SP services should prioritise community members’ needs across different aspects such as poor housing, mental health, debt, loneliness, unemployment and lifestyle conditions. *“We need a variety of services as we have different needs. Government should invest in the third-party sector, let it develop its capacity in local communities”* (EbE1). They also suggested providing subsidised transportation tickets for vulnerable people to ensure they can access services without being hindered by financial difficulties. “*Let’s think about practical solutions for transportation barriers; maybe subsidised ticket for deprived people is a key”* (EbE3).

Similarly, presenting concrete statistics and figures to funders and NHS commissioners about the effectiveness of SP programmes can demonstrate the significant impact and value for money of community activities. “*It did all the impact on the return on investment for every £1.00 invested there has been a £2.16 of additional benefit in terms of reduced costs to the NHS”* (CP2). The important role of motivators was also mentioned by some of the service providers as a facilitating factor for passing on information to different stakeholders of the SP programme. “*Information should be passed on to people in GP surgeries; maybe receptionists should take this responsibility”* (CP1).

#### Theme 2: applying appropriate payment mechanisms

In addition to training opportunities, LWs and CPs emphasised the need for their salaries/income to reflect their level of knowledge, skills and the amount of time and effort they dedicate to their work. LWs explained that while their salaries are reimbursed by the NHS, limited financial resources make it challenging to provide additional financial incentives to ensure retention in the workplace and maximum effort in delivering services. “*We need additional funding to take referrals from a wide range of health and social service agencies and work closely with community organisations; our tasks are not limited to referring clients, it is actually evaluating people’s needs and providing them personalised care”* (LW2). Similarly, concerns were raised by CPs who were dissatisfied that their salary and precarious working conditions did not reflect their skills and the need for their services to develop in response to community needs. *“Employment prospects, yes. Actually, again, at the meeting we were lots of service providers who thought the pay is rubbish, there is no support for the funding and all that kind of stuff”* (CP4).

#### Theme 3: research to evaluate users’ needs and expectations

Most of the study participants highlighted the significant role of research studies in defining output indicators and evaluating health impacts to ensure that users are referred to appropriate services and receive suitable support tailored to their needs. “*We see several studies exploring system’s barriers but honestly no practical solutions are developed through group efforts”* (CP2). The necessity for developing a unified measuring outcomes form was also emphasised by some of the CPs to ensure continuous monitoring of users’ experience throughout their SP journey. “*We should give people what they want; so, we need to be aware of their perspectives, their needs, their expectations and values”* (CP5).

Accordingly, most of the participants agreed that without research, significant progress in improving community well-being would be difficult to achieve. “*Up to date statistics on service users’ interest provide a potential to make future improvements in services”* (NHS3). Participants also stressed the importance of sharing research findings with all stakeholders in clear, accessible language, across easy-to-use communication platforms. This idea would create an accessible database for the optimisation of patient referrals, ensuring they have access to the most appropriate services based on their current concerns and experiences. *“Sharing decision-making among all team members is our ideal; but to get that purpose we need updated data around different perspective, belonging to different partners”* (CP2).

#### Theme 4: designing an inclusive education

Creating suitable conditions for inclusive education about SP and community well-being activities across all stages of life, from school to work, was identified as a crucial solution by service providers. “*Teaching people about their social needs gives them confidence in looking for right solutions”* (NHS1).

LWs emphasised the importance of prioritising members of the general public in decision-making processes to empower them to actively participate in promoting their health and well-being. “*Who is going to know their own wellbeing better than the people, but it is always decided by everyone else, isn’t it?”* (LW4). They also emphasised the significance of public sector workers cooperating between employees from different departments of public services to cultivate a comprehensive health culture within the community. *“All people who work in nurseries, schools, colleges and universities should get together with all of the people who may need the service”* (LW3). One CP mentioned co-design workshops as ideal settings for inviting people to actively participate in integrated groups, where their awareness towards population health and its determinants will develop. *“After six months of the project, we might invite them to creative workshops, which is not about targeted people with mental health disorders, but they know me as a musician who is always going to be there”* (CP3).

- **Meso level (organisational and interpersonal context):** this level pertains to organisational practices and structures, such as service delivery models, inter-organisational collaboration and the role of professionals within these settings.

#### Theme 5: constructive collaboration

NHS commissioners emphasised the key role of LWs as care navigators who have the responsibility to address non-clinical needs of the population and provide thorough support by connecting individuals with relevant services. They believed that embedding LWs within GP surgeries provides an opportunity for them to work as integral members of a multidisciplinary team. This makes them inseparable members of PCNs and subsequently increases awareness of the significant role of the SDH and the effectiveness of CPs in delivering community well-being activities to address a wide range of health needs. “*When GPs understand the usefulness of social prescribing services, they will recognize them as an important part of the health system*” (NHS3).

A key strategy to facilitate knowledge sharing is attendance at face-to-face meetings, social events and group discussions. “*We can share ideas in group meetings; everyone will be informed of our expectations, what challenges exist, and how to mitigate problems interactively”* (NHS1). The role of NHS commissioners in organising joint events was also highlighted as a key to get continuous feedback from all stakeholders and share information between different partners of the programme. “*Healthcare practitioners need to see SP services easy to use. This cannot be achieved unless all key stakeholders are informed from the beginning of the programme”* (CP5).

#### Theme 6: increasing the number of well-trained LWs and CP well-being workers

Most LWs agreed on the necessity to have access to training workshops and career progression opportunities. They acknowledged that a skilled LW needs to be trained about empathetic behaviour, listening skills and gaining people’s trust to act effectively as a counselling coordinator. *“Every day we deal with lots of mental health cases; so, to manage our interactions with them effectively we’d need further training to feel confident in our work”* (LW3). LWs also stressed the importance of ongoing supervision of their job activities by informed and capable managers to foster trust among support workers and promote a culture of teamwork. “*Where SP tasks go beyond our capacity, we need to benefit from clear instructions provided by managers” (LW4*).

CPs also noted that, as they often employ freelance staff, they need to resource appropriate supervision, reflective practice sessions and their workforce requires skill development. “*By working more collaboratively CPs felt some of these challenges could be addressed”* (CP1).

Additionally, LWs believed that by delivering collaborative information sharing to CPs, NHS commissioners, GPs, funders and EbEs about the requirements for the implementation of SP, it would enhance the usefulness, informativeness and effectiveness of the referral system. *“If they receive necessary information, they will understand the process easily, this knowledge leads to an increase in the quality of the referral system”* (LW2).

#### Theme 7: using the potential of capable IT technical support

Some health and social care professionals mentioned that all staff involved in the SP system are eager to receive up-to-date and accurate information about the impact of SP activities on peoples’ health conditions. They added that connecting information between different stakeholders involved in SP can enhance the healthcare delivery system’s ability to effectively serve communities by facilitating information sharing in different settings. The jurors also emphasised the critical role of an IT support system capable of integrating benefits, housing, legal and health information within the community. They believed that such a comprehensive information system could support personalised counselling and patient-centred care, aiming to promote health improvement, achieve well-being goals and effectively manage mental health conditions. They also believed that the current ‘tick-box’ approach to referrals is unlikely to effectively identify those most in need. They focused on the fact that having a top-down or tick-box approach will avoid referrers from managing the principles of co-ownership by the communities who seek help. “*We need to know more about IT and analytical skills to better work with the system*” (LW5).

#### Theme 8: redesigning the information system

Developing an integrated, holistic information system and an updated database was one of the important solutions that was emphasised by some of the study participants. *“Maybe the databases need to be more relevant and up to date”* (LW5). A group of CPs agreed that dealing with numerous directories in different formats causes significant challenges in accessing the necessary information effectively. “*I’m being sent numerous directories and databases. Goodness knows how many databases and I don’t suspect any of them are up to date*” (CP1). They suggested the need to organise training courses focused on digital skills and the development of a more proficient use of information systems. *“Maybe there is a training need; otherwise, they just seem like a total waste”* (CP3). Most of the CPs highlighted the importance of gathering various users’ opinions on the shortcomings of the existing information system and proposing solutions to address these issues. *“We see some gaps in the system; why not revisiting the system based on our experiences”?* (CP2). Most of the attendees mentioned the lack of access to GP Emergency Medical Services Information System (EMIS) as a major barrier within the system. *“We don’t have access to EMIS; we found not everything is on EMIS or things aren’t worded, or they are worded in a particular way, and you don’t get all the information even if you were to sit and go through someone’s record on EMIS*”.

#### Theme 9: determining consistent quality indicators in achieving SP objectives

One of the important indicators in evaluating the quality of services is continuity of care to ensure its effectiveness on people’s health and well-being. Evaluating users’ health condition at different time intervals through periodic follow-ups by LWs was recognised as one of the strategies for the issue. *“Sometimes GPs or nurses are saying we are going to send this over to social prescribing but the person isn’t ready to accept the help”* (LW2).

Study participants further explained that to ensure service standards are inclusively met it is necessary to understand whom the clients are, what their needs are and how they prefer services to be delivered. *“Knowing how to establish mutual partnerships with local businesses to support community groups in a sustainable way is of great importance”* (LW5).

One of the NHS commissioners highlighted LWs as crucial in identifying existing gaps in local community services and advocating for investment from commissioners and local agencies into the development of new activity groups. “*A link worker is a referral facilitator who is able to increase understanding between PCN staff and local communities*” (NHS4). Another participant noted that simplifying referral procedures and developing impact indicators should be facilitated through engagement with multiple stakeholders. *“To ensure an ongoing development of the programme, suitable monitoring system with clear evaluation criteria are crucial”* (NHS2). Inconsistencies in the evaluation process, due to a lack of integrated indicators, were also identified as significant barriers to achieving SP goals across different geographical regions. “*Making our own referral forms, that was the example you gave, I was just thinking we can also learn from each other’s systems. Is that something that you can do on your own?”* (CP5).

- **Micro level (individual context and experiences):** the micro level focuses on the individual experiences of service users, LWs and CPs, emphasising their personal interactions, needs, and responses to SP.

#### Theme 10: using the potential of hubs

The majority of EbEs believed that the SP system should foster an actively listening culture by responding to an individual’s needs with empathy and prioritising and advocating for community-led decision-making. They viewed co-production as essential, stressing the need for a language that resonates with various stakeholders. Building trust and incorporating lived experiences were highlighted as crucial prerequisites for successful co-production. “*Involving more people with a diverse range of lived experience is like keeping them in the driving seat*” (EbE2). Attendees declared that service users should work alongside LWs to co-produce an effective SP system. “*Co-production is about asking for our opinions, knowing that our ideas are heard in the system design and implementation”* (EbE5). They identified SP hubs as key connectors between individuals and community services, facilitating access to non-medical support and providing venues for interaction, collaboration, and teamwork. “*Hubs are important places to ensure appropriate access to non-medical support and a venue for getting together, talking to each other and doing team works”* (EbE1). They also highlighted the important role of hubs in promoting a person-centred approach, fostering human interaction and offering empathetic emotional support to improve health outcomes and user’s satisfaction.

#### Theme 11: defining appropriate criteria for referring people to SP services

Some of the participants devised a model called ‘CHAT’ (Clinical, Health, Assessment, Triage) as a mechanism for signposting individuals to suitable services based on their mental health and social care needs. In fact, they highlighted the need for SP to be normalised and suggested integrating it into a current health system’s preliminary assessment. To help the realisation of this approach, some of the EbEs proposed a dual-triage system that evaluates an individual’s physical health needs in addition to their mental and social healthcare needs. “*Signposting every person to something that gives them hope and value is the end goal of the CHAT model”* (EbE5).

CPs emphasised the need for a set of explicit and transparent indicators, in the form of referral guidelines, to ensure appropriate and integrated referrals to SP services. *“There are different referral categories, like mental health issues, loneliness, poor housing, depression and anxiety, anger, dementia and even issues associated with long-term diseases. Referrers need clear criteria to act seamlessly and in consistent order”* (CP3). Study participants also noted that there is a lack of consistency in referrals between different GP practices; this is a significant issue that happens due to varying reasons for referral and unclear referral procedures. One CP added that self‐referral is accepted in some GP surgeries but prohibited in others. No clear adherence to a unified process and subsequent variations in referrals were two main reasons for such inconsistencies. “*To my knowledge, there is no uniform, and standardised referral practices”* (CP1).

## Discussion and conclusion

### Summary of key findings

This study identified several key challenges and potential solutions related to the implementation of SP. The primary difficulties involved issues surrounding financial sustainability, the adequacy of the workforce (particularly LWs), inconsistencies in referral processes and the need for a more integrated system. Financial constraints, both for SP initiatives and community-based organisations, were frequently cited as the most pressing concern. In addition, participants emphasised the importance of a well-trained workforce, particularly LWs, who play a critical role in connecting individuals to non-clinical services. Furthermore, the study found a need for more effective communication and coordination between PCNs, local authorities and voluntary, community and social enterprise sectors to ensure sustainable, integrated services. Finally, a key finding was the necessity for an integrated information system to streamline referrals, track outcomes and facilitate communication across sectors.

### Comparison to existing literature

A key challenge identified by participants in this study was the financial sustainability of SP. Participants highlighted the importance of directing adequate funding to preventive care and addressing SDH. Existing literature supports these concerns, emphasising the need for long-term funding to ensure the financial viability of both the NHS and VCSE sectors.^19^,^21^ Furthermore, studies suggest that expanding preventative services leads to reductions in morbidity and mortality, particularly regarding chronic diseases and mental health.^22^ Effective management, leadership and collaboration between primary and secondary care are also needed to secure sustainable funding and resources for non-clinical needs.^19^

The funding of PCNs, which cover LW salaries and training costs, is an essential part of financing SP services.^23 24^ However, PCNs do not fund community resources to which LWs refer patients, requiring collaboration between NHS and local governments for VCSE support. This collaborative funding model ensures resource availability but requires additional funding to prevent sustainability issues due to increasing referrals.^25^ To address this, innovative funding models, such as leveraging private sector investment, are suggested in the literature.

Participants also emphasised the need to empower social support services through the VCSE sector, with NHS budgets allocated to finance local initiatives that offer non-clinical care. However, the growing demand for VCSE services calls for more financial resources. The need for long-term funding and equitable relationships between the NHS and VCSE sector was identified as crucial for sustainability. Additionally, an appropriate budget-pooling strategy between the NHS and local governments is proposed as a sustainable funding solution for SP initiatives.^20 21^

The role of LWs in reducing GP workload, delivering personalised care and addressing health issues was also noted by participants. Literature supports this, stating that training LWs in peer mentoring, communication and clinical supervision is essential for their effectiveness.^26^ Research by Wildman *et al* and Hayes *et al* suggests that well-trained LWs can build trust with service users and improve health outcomes, while a lack of training poses barriers.^21 27^ Training GPs and other professionals in SDH is also crucial for improving health outcomes.^28^ Moreover, a standardised evaluation system and improved information systems to track SP referrals and outcomes are necessary for enhancing the effectiveness of services.^27 29^

Participants discussed the use of community hubs to meet the social and emotional needs of individuals. Community hubs provide a variety of non-clinical services and can reduce the demand on GPs, supporting primary care.^30^ These hubs are part of a community-led SP model that involves various providers and local organisations.^31^

Human resource challenges, such as inadequate pay and support, were also mentioned. Long-term funding is seen as essential for providing employment stability. Additionally, unclear referral processes hinder timely and appropriate service delivery. Correct referrals are crucial as inappropriate ones increase costs.^32^ Literature suggests that a good connector between service users and local support services is necessary for improving health outcomes. Standardised outcome measures, such as the Warwick-Edinburgh Mental Wellbeing Scales, can assess SP effectiveness.^27^

Technical issues related to information systems were highlighted. Participants stressed the importance of well-trained information technologists (ITs) to support SP systems. Digital technologies can enhance service delivery by improving communication and providing on-demand services.^33^ However, challenges related to system usability and security underscore the need for competent IT professionals to ensure effective use of technology.^34^ Specialised training for SP staff is essential to empower them in using digital systems effectively.^35^

Effective partnerships and multidirectional collaborations between local communities, health and social care sectors, and funding organisations were also seen as vital. Inappropriate interactions between stakeholders can create gaps in service provision, while multidisciplinary team meetings can improve mutual understanding and collaboration.^33^ Building connections between PCNs and the VCSE sector is crucial for SP programme success.

Participants emphasised the role of trust-building in SP systems, especially with vulnerable groups. Ensuring adequate time for LWs to engage with service users and empowering LWs as connectors can foster long-term, sustainable communication.^35^ Integrated information systems for SP are also needed to facilitate sharing data across stakeholders. Literature suggests that such systems, when linked with electronic health records, can improve service quality and track referrals effectively.^36 37^

Finally, participants agreed that an evaluation system is necessary to ensure SP service quality. Literature supports the use of qualitative methods to assess user experiences and improve services.^32^ Ongoing research is also crucial for understanding user needs and expectations, and sharing findings with all stakeholders can enhance the accessibility of SP services.^38 39^

### Methodological critique

While this study offers valuable insights into the implementation of SP, there are some limitations in its methodology that must be acknowledged. The participatory approach used in the study was beneficial for gathering a broad range of perspectives, but lack of detailed, longitudinal data on the outcomes of SP initiatives means that the effectiveness of the proposed solutions remains theoretical. While the proposed solutions in this study are informed by evidence and participant perspectives, their implementation requires further evaluation and feasibility testing.

### Implications for research, practice and policy

In terms of research, it is suggested that future research explore more in-depth the long-term impact of SP on health outcomes, particularly in relation to mental health and chronic conditions. There is also a need for studies that evaluate the effectiveness of different funding models, training programmes for LWs and integration strategies between healthcare systems and VCSEs. Additionally, evaluating the success of integrated IT systems in improving referral processes and data sharing would contribute valuable knowledge.

On a practical level, our study suggests that greater attention should be paid to ensuring consistent and standardised referral processes and adequate training for LWs. There should also be a focus on developing strong, sustainable partnerships between healthcare providers, local authorities and the VCSE sector. Community hubs could be expanded as a key strategy for reducing GP workload and meeting the social and emotional needs of patients. Additionally, empowering LWs with more comprehensive training and resources is vital for ensuring they can perform their roles effectively.

Regarding policy, policymakers should prioritise funding mechanisms that support both healthcare and non-clinical services. Collaborative models of funding, pooling resources between the NHS and local governments, are essential for addressing the wider needs of the community. Moreover, clear standards and guidelines for referral processes should be established, and integration between health and social care systems must be encouraged. The development of an integrated information system to enhance communication and coordination across sectors is also critical for improving the implementation of SP services.

### Conclusions

This study highlights the key barriers to the implementation of SP, including financial sustainability, workforce training and coordination between sectors. By focusing on the practical aspects of SP, such as effective referral processes, funding models and workforce development, we offer insights into how to overcome these challenges. The findings underline the importance of collaboration, integration and long-term investment in SP services. Addressing these issues will be crucial for the future success of SP as a strategy to improve community health and well-being. Policymakers and healthcare providers must take these challenges into account to ensure that SP can be effectively scaled and integrated into health systems.

## supplementary material

10.1136/bmjopen-2024-094522 online supplemental file 1

## Data Availability

All data relevant to the study are included in the article or uploaded as supplementary information.
