# Supplementary material for: Co-designing implementation strategies for social prescribing in Lancashire and South Cumbria: a qualitative study with a participatory approach
Source: BMJ Open. 2025 Apr 2;15(4):e094522. doi: 10.1136/bmjopen-2024-094522 (PMC11966943; doi:10.1136/bmjopen-2024-094522)
Supplement: online supplemental file 1 [file bmjopen-15-4-s001.docx]

**Days of the Citizen’s Jury: 3^rd^ and 4^th^ of November**

Duration: 10:30 – 16:00

- Main aim of Day 1:

People will understand more deeply the range of issues affecting all stakeholders involved in social prescribing and be able to critically interrogate barriers and opportunities. They will have started to formulate ideas for the future.

- Main aim of Day 2:

Jurors will build upon their learning from day 1 and agree a series of recommendations for the future which will be presented to the room to ensure a collective understanding and buy-in for the future.

| **Day 1** | | |
| --- | --- | --- |
| Time | Exercise | Resources |
| 09:30-10:30 | Registration/ filling forms | Name badges  Consent forms  Demographic forms |
| 10:30-10:40 | Introduction to the project objectives and its achievements so far  **Prompts:**   - What were our study objectives? - What we have done so far? - What we have learned so far? | Slide  Video clip  Presenter (PI of the project) |
| 10:40-10:50 | A brief explanation about the day agenda and the method of citizen jury which is going to be applied.  **Prompts:**   - What are going to be done during these two days? - What is expected from participants? - What is the role of witnesses, jurors, and other participants? | Slide  Presenter (facilitator) |
| 10:50-  11:00 | A creative pause (an entertaining program) to refresh attendees at the beginning of the day. (before starting the presentations)  **Prompts:**   - Some musical/ art creativities (Sue, Jenny and Emma idea) | Depending on the selected program |
| 11:00-11:10 | First presentation by a **witness** from community providers  **Prompts**   - provide enough information / basic information about the subject in each area by her based on her expertise on her perspective on the issue/ personal experience and study findings that have been gained so far - ensure that jurors have access to a range of relevant opinions and evidence, which they can then scrutinise and synthesise with their own views to form collective recommendations - provide evidence and/or advocate for particular positions before being cross-examined by jurors | Digital voice recorders  Slide  Presenter |
| 11:10-11:30 | Q & A: Question and ask  **Prompts:**   - [jurors are able to question](https://participedia.xyz/method/566) witnesses, or even formulate questions together in small groups - Clarify all presented issues and topics - Be ready to digest all given information and conclude what messages they had taken from the presentations | Digital voice recorders |
| 11:30-11:40 | Second presentation by a **witness** from NHS coordinator  **Prompts**   - provide enough information / basic information about the subject in each area by her based on her expertise on her perspective on the issue/ personal experience and study findings that have been gained so far - ensure that jurors have access to a range of relevant opinions and evidence, which they can then scrutinise and synthesise with their own views to form collective recommendations - provide evidence and/or advocate for particular positions before being cross-examined by jurors | Digital voice recorders  Slide  Presenter |
| 11:40-12:00 | Q & A: Question and ask  **Prompts:**   - [jurors are able to question](https://participedia.xyz/method/566) witnesses, or even formulate questions together in small groups - Clarify all presented issues and topics - Be ready to digest all given information and conclude what messages they had taken from the presentations | Digital voice recorders |
| 12:00-12:10 | Third presentation by a **witness** from link worker  **Prompts**   - provide enough information / basic information about the subject in each area by her based on her expertise on her perspective on the issue/ personal experience and study findings that have been gained so far - ensure that jurors have access to a range of relevant opinions and evidence, which they can then scrutinise and synthesise with their own views to form collective recommendations - provide evidence and/or advocate for particular positions before being cross-examined by jurors | Digital voice recorders  Slide  Presenter |
| 12:10-12:30 | Q & A: Question and ask  **Prompts:**   - [jurors are able to question](https://participedia.xyz/method/566) witnesses, or even formulate questions together in small groups - Clarify all presented issues and topics - Be ready to digest all given information and conclude what messages they had taken from the presentations | Digital voice recorders |
| 12:30-12:40 | A creative pause (an entertaining program) to refresh attendees at the beginning of the day  **Prompts:**  Some musical/ art creativities (Sue, Jenny and Emma idea) | Depending on the selected program |
| 12:40-12:50 | Coffee and Break | |
| 12:50-13:00 | Forth presentation by a **witness** from service user  **Prompts**   - provide enough information / basic information about the subject in each area by her based on her expertise on her perspective on the issue/ personal experience and study findings that have been gained so far - ensure that jurors have access to a range of relevant opinions and evidence, which they can then scrutinise and synthesise with their own views to form collective recommendations - provide evidence and/or advocate for particular positions before being cross-examined by jurors | Digital voice recorders  Slide  Presenter |
| 13:00-13:20 | Q & A: Question and ask  **Prompts:**   - [jurors are able to question](https://participedia.xyz/method/566) witnesses, or even formulate questions together in small groups - Clarify all presented issues and topics - Be ready to digest all given information and conclude what messages they had taken from the presentations | Digital voice recorders |
| 13:20-14:00 | Lunch time |  |
| 14:00-15:15 | - Visioning activity   **Prompts:**   - Participants will be asked to think of future and imagine a positive vision for 10 years’ time about an optimal SP system you’d like to benefit from - Participants will be asked to visualise the desired social prescribing system from different aspects - each jury member is going to be given five minutes on their own with pen and paper sketching out their vision (or writing key thoughts). - they will join in small groups to discuss and share their visions - they will be asked to choose three words that think best describe their vision - The words will then be compiled to make a word cloud |  |
| 15:15-16:00 | End up the first session  **Prompts:**   - Review what had been done | |
| **Day 2** | | |
| 10:00-10:30 | Coffee and tea-warm up | |
| 10:30-11:15 | Deliberation  **Prompts:**   - jurors will discuss in-depth the evidence they have heard - jurors will work towards developing a set of recommendations or making a collective decision - jurors will work in small groups and finally in a whole jury |  |
| 11:15-12:00 | Reflection  **Prompts:**   - jurors will collect information for possible recommendations |  |
| 12:00-13:00 | Public Forum  Prompts:   - developing a public forum by jurors |  |
| 13:00-13:45 | Lunch | |
| 13:45-14:05 | Presenting the forum to policy makers by jurors | |
| 14:05-14:45 | Discussion and final decision making | |
| 14:45-15:00 | End up the event | |
