# Supplementary material for: Co-designing implementation strategies for social prescribing in Lancashire and South Cumbria: a qualitative study with a participatory approach
Source: BMJ Open. 2025 Apr 2;15(4):e094522. doi: 10.1136/bmjopen-2024-094522 (PMC11966943; doi:10.1136/bmjopen-2024-094522)
Supplement: Supplementary file 2 [file bmjopen-2024-094522.reviewer_comments.pdf]

PEER REVIEW HISTORY

BMJ Open publishes all reviews undertaken for accepted manuscripts. Reviewers are asked to complete a checklist review form and are provided with free text boxes to elaborate on their assessment. These free text comments are reproduced below.

ARTICLE DETAILS

Title (Provisional)

Co-designing Implementation Strategies for Social Prescribing in Lancashire and South Cumbria: A Qualitative Study with a Participatory Approach

Authors

Rafiei, Sima; Honary, Mahsa; Mezes, Barbara; Flowers, Susan

VERSION 1 - REVIEW

**Reviewer**	**1**
**Name**	**Macfarlane, Anne**
**Affiliation**	**University of Limerick, Graduate Entry Medical School**
**Date**	**25-Nov-2024**
**COI**	**None**

Thank you for the opportunity to review this paper about using a co-production process to improve the implementation of social prescribing. This is an important area of research in terms of the focus on social prescribing in primary healthcare and the focus on co-production as a way of generating knowledge to address translational gaps in our field. The authors have used a specific co-design methodology from the Hasso Plattner Institute of Design and have completed valuable work with multiple stakeholders. However, the write up of the work needs significant attention and substantial revisions with particular attention to concepts, framing of the work, methodological detail and data synthesis.

Introduction

More work is needed to clarify and refine the concept of co-production/co-design. Indeed, different terms are used in the paper: co-production, co-design and participatory framework. A formal definition is only provided for co-production. It is well known that different terms are used in this field but this paper shows that, in the main, they refer to similar values and processes (see How does integrated knowledge translation (IKT) compare to other collaborative research approaches to generating and translating knowledge? Learning from experts in the field - PubMed Nguyen et al., 2020). For advancing knowledge about co-production, it is good practice to specify terms and definitions in this paper more carefully. My suggestion is to make it clear that there is heterogeneity and interchangeable

use of terms and explain that, in this paper, term X is being used and here is the definition of that term.

Consider framing the focus of the work more explicitly as a knowledge translation issue and summarise literature about the role of co-production to support gaps in knowledge translation. Perhaps say here (or in methods) what range of methods were considered to support the co-design process and why Hasso Plattner Institute of Design was selected.

Expand the argument that there is a need for looking at root causes of known challenges rather than merely tackling those challenges without going through that analytic step.

Adding a clear research question to the end of the introduction would strengthen the paper.

Methods

Was this study a participatory health research study (Jagosh et al)., 2012 Uncovering the Benefits of Participatory Research: Implications of a Realist Review for Health Research and Practice - JAGOSH - 2012 - The Milbank Quarterly - Wiley Online Library i.e., was the idea for it developed and taken forward between academic and community partners or is an academic driven study that is using participatory methods to progress the work? Either is fine but it is important that this is clear to readers.

Did all 24 participants attend all four workshops?

What sampling approaches were used e.g., purposeful sampling?

Was displaying information about the study sufficient for recruitment or was this combined with other more active forms of outreach to potential participants?

How long were the workshops?

More information is needed about the conduct of the workshops e.g., make it clear what steps of the selected co-design methodology from the Hasso Plattner Institute of Design were progressed in which workshops.

How feasible was it to audio record the workshops given that (I assume) there were interactive with lots of small group discussion?

There is no information provided about the procedures used in the Citizen Juries and how they were analysed and distilled vis a vis the findings from the four workshops. This is problematic given that findings are reported from that part of the work in the results section.

What steps were taken to enhance the quality and rigour of the reported thematic analysis?

Results

The results sections is generally comprehensive but hard to follow and somewhat overwhelming. More detail is needed in some places and more synthesis would be extremely valuable to offer readers a more in-depth understanding of the findings.

It needs to be much clearer what the shared and differential findings were between participant groups in the fishbone diagrams. A paragraph/visual synthesising this would be useful.

Provide information about the procedures used in the Citizen Jury

Is it eleven solutions or themes?

Expand the analysis of these eleven solutions /themes to offer a higher level conceptual analysis of what the data are about e.g., map the findings onto a macro-meso-micro level framework or onto a relevant implementation theory.

Discussion

This section was very long and unwieldy. It would strengthen this section considerably to re-organise the material into clear sections: (i) high level summary of key findings (ii) how these findings are similar to/expand the existing literature (iii) methodological critique (iv) implications for research, practice and policy and (v)short conclusion to spell out the key learning and 'take home' message

For this re-write, it would be important to ensure that the narrative is about findings about implementation of social prescribing (the study focus) rather than the rationale for social prescribing of attending to social determinants of health (the background doing the research in the first place). In the present draft, these issues are mixed up.

Additional points

There are multiple abbreviations used throughout the paper and it becomes very hard to keep track. Re-examine which terms really need abbreviations and refine the amount being used. I would suggest not using abbreviations for the stakeholder groups.

Cross check the use of reference 9 in the paper as it does not always seem to align with the content.

**Reviewer**	**2**
**Name**	**Langley, Joe**
**Affiliation**	**Sheffield Hallam University, Lab4Living**
**Date**	**23-Dec-2024**
**COI**	**None**

Apologies (in advance) for the brevity of my review. I am super interested in the topic area. This is not an excuse - but hopefully some explanatory context.

IMO - this is relevant work, filling a gap, with a waiting audience. BUT... needs refining.

Subject areas (SP), super important. Implementation is identified as 'the' challenge in focus. It may be worth considering whether the challenges are 'implementation' or 'adoption'? I think there is a difference. EG things might be implemented but perhaps people are refusing to use them or unaware of them? An issue (for stuff that is already implemented) may be more about awareness raising, publicity, education... etc.

Co-design, highly relevant method to address either issue.

Gaining consent is mentioned but there is no reference (I may have missed it) to any ethical approval process or ethical approval being granted. This may be OK - but it needs to be addressed. Either you have ethical approval (if so, say when, who, provide ref number). If not, justify it.

Love the reporting of the co-design - clear. I think it would help to describe the disciplinary expertise/experience of the researchers and/ facilitators involved. May also help to provide references (and/or examples) for some of the more design methods (5 why's, crazy 8 etc). Personally, I'd also appreciate some context about how you 'staged' the workshops; where were they? what time of day? who attend each workshop? how did you create conditions for people to come together and 'design'? These details are often missing/lost but so important for successful co-design.

The main point for improvement is that there is a gap in our mutual understanding of 'implementation' which is illustrated in multiple ways throughout the article, even from the title.

The title claims that the work described co-designed *successful* implementation strategies. How do you know they are *successful*?

The abstract claims that you develop *practical* solutions. What is practical about them? They are solutions ideas, but there is no practical detail about how you would deliver adequate funding for SP, nor for implementing/building/sustaining a 'hub'. There is no practical detail about how you would fund, train, support LWs or CP wellbeing working (all of which would need funding) ... etc etc...

This seems to betray a very superficial knowledge of what is really involved in implementing any of these *ideas*. I have led a co-design and implementation piece of work relating to community Parkinson's services in SW England, and Community stroke services in SY. And

other cases. We generated ideas in similar processes to these. We then co-created the details (education/training information, SOPs, IT FAQs, implementation roll out plans, on-going evaluation and assessment schedules, information governance policies and data exchange agreements, funding approvals...). I know the level of practical detail involved to actually implement something from an idea to something people are actually using. What you describe is not this....

What you have, are co-designed solution ideas that are backed up by, and related to evidence from other studies. This makes them evidence based ideas, informed by experience. But this does not (yet) make them practical or successful. This does not diminish the importance and value of what you have done. But I would suggest you step back from such language a little, otherwise it would appear to be over 'egging' what you have actually done - which is still important

Your references to Implementation are thin. Of few. You pick up on the Goodyear-Smith article but this is for ethics committees, not for 'practical' solutions to SP.

Even the Campbell complex intervention development framework is out of date (see Skivington 2021 Complex intervention development) and totally inappropriate - it is based on a trials perspective of developing and evaluating interventions which co-design is almost the antithesis of.

There are so many implementation frameworks that could be useful and relevant to this work, from the field of implementation science, and also particularly from Knowledge Mobilisation. It needs more than me simply offering you a few to use. You need to gain a proper understanding of this area to make relevant references.

VERSION 1 - AUTHOR RESPONSE

Reviewer 1:

Comment 1: More work is needed to clarify and refine the concept of co-production/co-design. Indeed, different terms are used in the paper: co-production, co-design and participatory framework. A formal definition is only provided for co-production. It is well known that different terms are used in this field but this paper shows that, in the main, they refer to similar values and processes (see How does integrated knowledge translation (IKT) compare to other collaborative research approaches to generating and translating knowledge? Learning from experts in the field - PubMed Nguyen et al., 2020). For advancing knowledge about co-production, it is good practice to specify terms and definitions in this paper more carefully. My suggestion is to make it clear that there is heterogeneity and interchangeable use of terms and explain that, in this paper, term X is being used and here is the definition of that term.

Response to comment 1: Thank you for this insightful comment. We have revised the manuscript to clarify the relationship between co-production and co-design and ensure consistency in terminology. Specifically, we provided explicit definitions of co-production and co-design in the "introduction section", ensuring clarity in how these terms are applied in our study. We also acknowledged the interchangeable use of terms in the literature while specifying that we will use 'co-production' consistently to reflect the collaborative nature of the approach. Based on your comment, we incorporated references (including Nguyen et al., 2020) to justify our terminology choices.

Comment 2: Consider framing the focus of the work more explicitly as a knowledge translation issue and summarise literature about the role of co-production to support gaps in knowledge translation. Perhaps say here (or in methods) what range of methods were considered to support the co-design process and why Hasso Plattner Institute of Design was selected.

Response to comment 2: We have revised the manuscript to explicitly frame the study as a knowledge translation (KT) issue in the "Introduction", emphasizing how co-production serves as a KT strategy to bridge the gap between research and practice. We also summarised relevant literature on co-production and its role in KT, incorporating key references (e.g., Nguyen et al., 2020; Boyle & Harris, 2009; Dunston et al., 2009). Finally, in the "methods" section, we clarified different choices by outlining the range of participatory frameworks considered (e.g., IKT, CBPR, EBCD) and justified the selection of the Hasso Plattner Institute of Design's approach in this section. We briefly explained the benefits of the latter approach, particularly its iterative nature, stakeholder-driven process, and alignment with participatory research principles.

Comment 3: Expand the argument that there is a need for looking at root causes of known challenges rather than merely tackling those challenges without going through that analytic step.

Response to comment 3: We added an explanation in the "Introduction" that explicitly states why root cause analysis is necessary for social prescribing implementation. Related references were also added.

Comment 4: Adding a clear research question to the end of the introduction would strengthen the paper.

Response to comment 4: We have revised the end of the "Introduction" section to explicitly state the study's research question: What are the root causes of the key challenges in

implementing social prescribing programs, and how can co-designed strategies be developed to address these challenges effectively?

Comment 5: Was this study a participatory health research study (Jagosh et al)., 2012 Uncovering the Benefits of Participatory Research: Implications of a Realist Review for Health Research and Practice - JAGOSH - 2012 - The Milbank Quarterly - Wiley Online Library i.e., was the idea for it developed and taken forward between academic and community partners or is an academic driven study that is using participatory methods to progress the work? Either is fine but it is important that this is clear to readers.

Response to comment 5: Thank you for this insightful comment. We have revised the "Methods section" to clarify the participatory nature of the study. Specifically, we now explicitly state that this study is an academic-led study that integrates participatory methods. This clarification ensures that readers accurately understand the study's methodological positioning.

Comment 6: Did all 24 participants attend all four workshops?

Response to comment 6: Thank you for this important clarification request. We have revised the "Methods" section to specify that each of the four co-design workshops had 6–7 participants, rather than all 24 participants attending every session. This ensures transparency regarding participant engagement and how stakeholder representation was distributed across the workshops.

Comment 7: What sampling approaches were used e.g., purposeful sampling?

Response to comment 7: To capture a variety of insights, we adopted a purposive sampling strategy by targeting specific stakeholder groups. These included experts-by-experience (EbEs), social prescribing link workers (LWs), community providers (CPs), and healthcare professionals. We deliberately varied the composition of participants across the four workshops, ensuring that each session included a mix of these stakeholder groups. This approach allowed us to incorporate a wide range of perspectives while also accommodating participants' availability and expertise.

Comment 8: Was displaying information about the study sufficient for recruitment or was this combined with other more active forms of outreach to potential participants?

Response to comment 8: In our study, displaying posters and flyers in public venues such as GP surgeries, community centres, libraries, and locations where social prescribing activities were offered formed the primary means of recruitment. However, this was supplemented by more active outreach methods to ensure a broader and more engaged participant pool. Specifically, the National Institute for Health and Care Research (NIHR) supported us during

the recruitment phase. This collaboration helped increase visibility and reach, facilitating engagement with a wider pool of potential participants.

Comment 9: How long were the workshops?

Response to comment 9: The length of each workshop was 2 hours including refreshment.

Comment 10: More information is needed about the conduct of the workshops e.g., make it clear what steps of the selected co-design methodology from the Hasso Plattner Institute of Design were progressed in which workshops.

Response to comment 10: More information was added to the “Methods” section to clarify how different steps of Hasso Plattner Institute of Design were conducted. In fact, each co-design workshop lasted two hours and was structured into five sections. In each of these sections, we progressively developed and implemented the five steps of the HPI Design Thinking methodology, ensuring that participants actively contributed to each phase. In the “Methods” section, we explained the breakdown of how the methodology was applied in each section of the workshops.

Comment 11: How feasible was it to audio record the workshops given that (I assume) there were interactive with lots of small group discussion?

Response to comment 11: To ensure we captured the discussions clearly, for each workshop, we used four voice recorders, one for each small group. This allowed us to capture the voices of all participants in each group with clarity, ensuring that we could later transcribe the discussions accurately for analysis.

Comment 12: There is no information provided about the procedures used in the Citizen Juries and how they were analysed and distilled vis a vis the findings from the four workshops. This is problematic given that findings are reported from that part of the work in the results section.

Response to comment 12: The reason for not providing extensive detail in the manuscript regarding the Citizen Jury process was primarily due to the word limit constraints for the manuscript. As a result, we focused on providing a concise summary of the findings. However, we recognise the importance of providing more clarity, and we would like to highlight that the authors plan to draft and publish a separate policy report that will provide an in-depth exploration of the Citizen Jury process, its methodology, and the analysis conducted.

Additionally, to help clarify the Citizen Jury process in the current manuscript, we have uploaded the agenda for the Citizen Juries as a supplementary file. This document provides a detailed breakdown of the two-day event, including the activities conducted, structure of the sessions, and the steps followed during the Citizen Jury process. However, to respond the

reviewer, we added more information about the procedures used in CJ and how we analysed related data.

Comment 13: What steps were taken to enhance the quality and rigour of the reported thematic analysis?

Response to comment 13: We used three strategies (including clear coding framework, regular team discussions, and member checking) to enhance the quality and rigour of the reported thematic analysis. Explanation has been added at the end of the section "data analysis".

Comment 14: It needs to be much clearer what the shared and differential findings were between participant groups in the fishbone diagrams. A paragraph/visual synthesising this would be useful.

Response to comment 14: Dear reviewer, we provided four distinct fishbone diagrams, each corresponding to one group of participants (e.g., experts-by-experience, link workers, community providers, and healthcare professionals). These diagrams were designed to visually represent the key issues identified by each group and allow for a clear comparison of their responses. Following each diagram, we included a brief explanation that highlighted the similarities and differences in the responses across the groups.

Comment 15: Provide information about the procedures used in the Citizen Jury

Response to comment 15: We provided detailed information about the procedures used in the Citizen Jury and added to the manuscript. Please also see the supplementary file (depicting the CJ-event agenda).

Comment 16: Is it eleven solutions or themes?

Response to comment 16: That is eleven themes. Thank you for mentioning; and I revised in the manuscript text.

Comment 17: Expand the analysis of these eleven themes to offer a higher-level conceptual analysis of what the data are about e.g., map the findings onto a macro-meso-micro level framework or onto a relevant implementation theory.

Response to comment 17: Thank you for suggesting Macro-Meso-Micro Framework for Thematic Analysis. We re-organised the themes to fit in Macro, Meso and Micro levels properly.

Comment 18: Discussion was very long and unwieldy. It would strengthen this section considerably to re-organise the material into clear sections: (i) high level summary of key findings (ii) how these findings are similar to/expand the existing literature (iii) methodological critique (iv) implications for research, practice and policy and (v)short conclusion to spell out the key learning and 'take home' message

For this re-write, it would be important to ensure that the narrative is about findings about implementation of social prescribing (the study focus) rather than the rationale for social prescribing of attending to social determinants of health (the background doing the research in the first place). In the present draft, these issues are mixed up.

Response to comment 18: We organised “Discussion” into 5 sections: key findings; comparison with the existing literature; methodological critique; and implication for research, practice, and policy, and a short conclusion.

Comment 19: There are multiple abbreviations used throughout the paper and it becomes very hard to keep track. Re-examine which terms really need abbreviations and refine the amount being used. I would suggest not using abbreviations for the stakeholder groups.

Response to comment 19: We removed unnecessary abbreviations throughout the manuscript and tried to refer to them as footnotes. Regarding abbreviations for the stakeholder groups, we need to refer to them at the end of each quote. Therefore, in the “Result” section these abbreviations still exist at the end of each quote.

Comment 20: Cross check the use of reference 9 in the paper as it does not always seem to align with the content.

Response to comment 20: You are completely right. Reference 9 refers to “Co-Production and Health System Reform” and we made necessary corrections.

Reviewer 2:

Comment 1: Gaining consent is mentioned but there is no reference (I may have missed it) to any ethical approval process or ethical approval being granted. This may be OK - but it needs to be addressed. Either you have ethical approval (if so, say when, who, provide ref number). If not, justify it.

Response to comment 1: Thank you for the comment. We added an explanation to the “Ethical Considerations” and mentioned that: "Throughout the project, we adhereded to the ethical standards set out in Lancaster University's Code of Practice and the research ethics guidelines specified by the research councils. Ethical approval was obtained through both the NHS Health Research Authority (HRA) and the Research Ethics Committee at Lancaster University. The ethics approval ID is 317313."

Comment 2: Love the reporting of the co-design - clear. I think it would help to describe the disciplinary expertise/experience of the researchers and/ facilitators involved. May also help to provide references (and/or examples) for some of the more design methods (5 why's, crazy 8 etc). Personally, I'd also appreciate some context about how you 'staged' the workshops; where were they? what time of day? who attend each workshop? how did you create conditions for

people to come together and 'design'? These details are often missing/lost but so important for successful co-design.

Response to comment 2: Thank you for your thoughtful and constructive feedback. I have added details about the workshop setting, specifying that sessions took place at the School of Management Science, Lancaster University, between September and October 2023, with both morning and afternoon sessions to accommodate participants. Additional details on participant composition, facilitation expertise, and co-design methods (5 Whys, Crazy 8s) have been provided, along with references where applicable. In fact, each co-design workshop lasted two hours and was structured into five sections. In each of these sections, we progressively developed and implemented the five steps of the HPI Design Thinking methodology, ensuring that participants actively contributed to each phase. In the "Methods" section, we explained the breakdown of how the methodology was applied in each section of the workshops. The detailed information is added to the "Methods" section and highlighted in green.

Comment 3: The main point for improvement is that there is a gap in our mutual understanding of 'implementation' which is illustrated in multiple ways throughout the article, even from the title. The title claims that the work described co-designed *successful* implementation strategies. How do you know they are *successful*? The abstract claims that you develop *practical* solutions. What is practical about them? They are solutions ideas, but there is no practical detail about how you would deliver adequate funding for SP, nor for implementing/building/sustaining a 'hub'. There is no practical detail about how you would fund, train, support LWs or CP wellbeing working (all of which would need funding). What you have, are co-designed solution ideas that are backed up by, and related to evidence from other studies. This makes them evidence based ideas, informed by experience. But this does not (yet) make them practical or successful. This does not diminish the importance and value of what you have done. But I would suggest you step back from such language a little, otherwise it would appear to be over 'egging' what you have actually done - which is still important.

Response to comment 3: We appreciate your insight regarding the distinction between co-designing solutions and demonstrating their practicality or success in implementation. To address this, regarding "Title & Abstract", we have revised the title to remove the term successful and ensure it more accurately reflects the study's scope. In the abstract, we have clarified that the study presents evidence-based, co-designed solution ideas rather than practical solutions, acknowledging that further steps are needed to assess feasibility and implementation. Furthermore, throughout the discussion, we have refined our language to avoid overstatement. Instead of claiming the solutions are practical or successful, we now emphasise that they are

co-designed strategies informed by participant insights and existing evidence. We explicitly note that while these solutions align with best practices and research findings, additional work is required to operationalize them, particularly in terms of funding, training, and sustainability.

Comment 4: Your references to Implementation are thin. Of few. You pick up on the Goodyear-Smith article but this is for ethics committees, not for 'practical' solutions to SP. Even the Campbell complex intervention development framework is out of date (see Skivington 2021 Complex intervention development) and totally inappropriate - it is based on a trials perspective of developing and evaluating interventions which co-design is almost the antithesis of. There are so many implementation frameworks that could be useful and relevant to this work, from the field of implementation science, and also particularly from Knowledge Mobilisation. It needs more than me simply offering you a few to use. You need to gain a proper understanding of this area to make relevant references.

Response to comment 4: We acknowledge that the Campbell framework was not the best fit for a co-design approach and have removed it. We now align our "Introduction" with more suitable implementation theories, emphasizing their relevance to co-design and social prescribing. In response to your comments, we have now explicitly justified our use of Knowledge Transfer as a key approach in our study. We chose KT because of its relevance to Co-Design & Social Prescribing; also, as it focuses on translating research into practice by ensuring that knowledge is co-produced, shared, and applied in real-world settings. Furthermore, given that social prescribing involves multiple stakeholders (e.g., healthcare providers, link workers, community organizations), KT provides a structured yet flexible approach to integrating diverse perspectives and ensuring practical applicability.

In terms of KT alignment with Implementation Science, we see KT approach aligning better with co-design by emphasising co-creation of knowledge, and the iterative adaptation of solutions. We appreciate your constructive feedback, which has significantly enhanced the clarity and rigor of our theoretical positioning.
